# Mapping twenty years of corn and soybean across the US Midwest using the Landsat archive

**DOI:** 10.1038/s41597-020-00646-4

**Published:** 2020-09-15

**Authors:** Sherrie Wang, Stefania Di Tommaso, Jillian M. Deines, David B. Lobell

**Affiliations:** 1 Institute for Computational and Mathematical Engineering, Stanford University, Stanford, CA USA; 2 Center on Food Security and the Environment, Stanford University, Stanford, CA USA

**Keywords:** Sustainability, Agriculture

## Abstract

Field-level monitoring of crop types in the United States via the Cropland Data Layer (CDL) has played an important role in improving production forecasts and enabling large-scale study of agricultural inputs and outcomes. Although CDL offers crop type maps across the conterminous US from 2008 onward, such maps are missing in many Midwestern states or are uneven in quality before 2008. To fill these data gaps, we used the now-public Landsat archive and cloud computing services to map corn and soybean at 30 m resolution across the US Midwest from 1999–2018. Our training data were CDL from 2008–2018, and we validated the predictions on CDL 1999–2007 where available, county-level crop acreage statistics, and state-level crop rotation statistics. The corn-soybean maps, which we call the Corn-Soy Data Layer (CSDL), are publicly hosted on Google Earth Engine and also available for download online.

## Abstract (summary)

**Table:** 

Measurement(s)	corn plant (sensu maize) • soybean plant (food source)
Technology Type(s)	satellite imaging of a planet • digital curation • supervised machine learning
Factor Type(s)	year of data collection
Sample Characteristic - Environment	area of cropland • agricultural field
Sample Characteristic - Location	Midwest Region

Machine-accessible metadata file describing the reported data: 10.6084/m9.figshare.12839777

## Background & Summary

The Midwestern United States is one of the most intensive agricultural areas in the world, producing over 33% of the world’s corn and 34% of the world’s soybeans^1^. The sustained productivity of this region requires an understanding of how its agronomic practices, ecological properties, and production outcomes have evolved through time, which in turn relies on knowing the location and extent of its two dominant crops over many years. Since 2008, the confluence of Landsat and other satellite imagery, training data from the Farm Service Agency, and scalable machine learning algorithms has enabled the United States Department of Agriculture (USDA) to create the Cropland Data Layer (CDL), which maps 108 crop types at 30 m spatial resolution across the 48 conterminous US states^2^. While it was created primarily to aid the USDA’s annual crop area estimates, CDL has enabled a multitude of downstream research and operational work in the past decade^3^, chief among them forecasting food production^4^, monitoring crop yields^5–9^, identifying agronomic practices^10–17^, and assessing ecological impacts^18–23^.

Going further back in time, however, CDL no longer covers the conterminous US or even the entire Corn Belt: in 2007, CDL mapped 21 states; in 2004, 11 states; in 2001, 8 states, and in 1999, 4 states^2^. Major barriers that impeded the creation of large-scale CDL in the early 2000s include a lack of computational power and the cost of satellite data, which at the time was purchased by the image^24^. Today, the entire Landsat archive is free and available^25^ as calibrated surface reflectance products^26^, and cloud computing platforms like Google Earth Engine have vastly decreased the time, complexity, and cost of training machine learning models on satellite imagery^27^. The past two decades of constraints and advances can be seen in the changing composition of CDL, summarized in Table 1; for instance, in the shift from primarily using Landsat imagery to ResourceSat imagery and then back to Landsat between 2004 and 2010, and from using an in-house maximum likelihood classifier to an external decision tree algorithm in 2006^4^.

**Table 1:** Comparison of the USDA’s Cropland Data Layer (CDL) versus Corn-Soy Data Layer (CSDL).

	Cropland Data Layer	Corn Soy Data Layer
Land Cover Classes	133 classes	Corn, soybean, other
Geographic and Temporal Coverage	1 state (1997–1998)	13 states (1999–present)
	4 states (1999)	
	6 states (2000)	
	8 states (2001)	
	18 states (2002)	
	9 states (2003)	
	11 states (2004–2005)	
	16 states (2006)	
	21 states (2007)	
	48 states (2008–present)	
Satellite Imagery	Landsat 5 TM (1997–2006, 2010–2012)	All available images from: Landsat 5 TM (1997–2012) Landsat 7 ETM+ (1999–present) Landsat 8 OLI (2013–present)
	Landsat 7 ETM+ (1999–2006, 2010–2012)	
	ResourceSat-1 AWiFS (2006–2010)	
	Landsat 8 OLI (2013–present)	
	Deimos-1 (2011–present)	
	UK-DMC 2 (2011–present)	
	ResourceSat-2 LISS-3 (2017–present)	
	Sentinel-2 (2017–present)	
Spatial Resolution	30 m (1999–2005)	30 m
	56 m (2006–2007)	
	30 m (2008–present)	
Temporal Frequency	Annual	Annual
Classifier	Peditor maximum likelihood classifier (1997–2005)	Google Earth Engine random forest classifier
	RuleQuest See5 decision tree (2006–present)	
“Ground Truth” Training Labels	June Agricultural Survey (1997–2005)	CDL 2008–present
	Farm Service Agency CLU (2006–present)	
	National Land Cover Data (1997–present)	
Validation	Subset of ground truth	CDL 1999–present NASS acreage estimates

The changes in CDL throughout the years reflect the technologies available at the time, while CSDL was created using the entire Landsat archive, a consistent machine learning algorithm, and uniform 30 m resolution.

If CDL were extended back in time, expanding our knowledge of where crops were grown across the Midwest, the longer time series would enable researchers to assess the impact of events prior to 2008 on agriculture in the Corn Belt at a finer spatial resolution than ever before. An example is the effect of the 2007 Renewable Fuel Standard on corn intensification, crop rotation, and water use, which has been studied through modeling of crop rotation probabilities^28^ but not yet via observed crop types. Longer crop type time series would also enable derivation of more robust and spatially detailed relationships between variables like crop rotation^10,11,29^, tillage^13,30^, water use^31^, flooding^32^, climate^33^, and in-season satellite imagery^7,8^ and outcomes like yields. For the studies cited, gaps in CDL prior to 2008 were one factor limiting their scope to recent years or a subset of states with long histories of CDL.

In this work, we created maps of corn and soybean across 13 states of the US Midwest back to 1999, using all available imagery from the Landsat archive and a consistent methodology across all years. These 13 states together produce 90% of US corn and soybeans, yet for the following state-years no CDL data currently exists: Iowa (1999), Indiana (1999), Nebraska (1999–2000), Minnesota (1999–2005), South Dakota (1999–2005), Kansas (1999–2005), Wisconsin (1999–2002), Missouri (1999–2005), Ohio (1999–2005), Michigan (1999–2006), and Kentucky (1999–2007). Even for years and states with data, CDL can vary in quality and resolution (56 m in 2006–2007). To fill these gaps and smooth inconsistencies, we used Google Earth Engine to train a random forest classifier on Landsat-derived harmonic regression features and CDL labels—simplified to corn, soybean, and one “other” crop class—from 2008–2018. We call this map the Corn-Soy Data Layer, or CSDL.

**Fig. 1:**
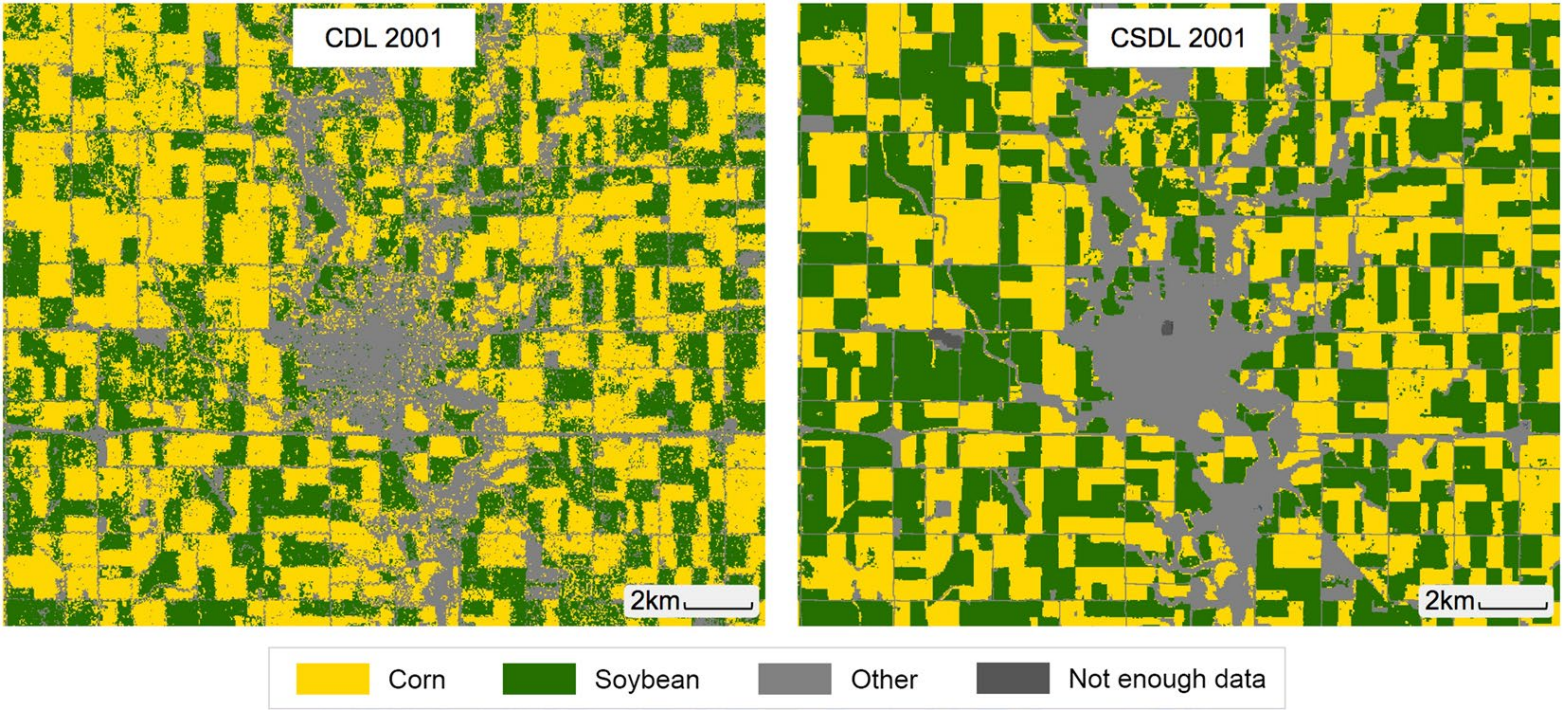
Corn and soybean classification around Webster City, Hamilton County, Iowa in 2001. The USDA’s CDL is shown on the left and our Corn-Soy Data Layer (CSDL) on the right.

To validate CSDL, we compared it (1) to CDL in the years and states where CDL is available, (2) to planted acreage from the National Agricultural Statistics Service (NASS) in all years and states, and (3) to rotation statistics from the Agricultural Resource Management Survey (ARMS) in 2001, 2005, and 2010. While each of these “ground truth” sources contain their own errors and uncertainties, when taken together the comparisons suggest that CSDL largely captures where corn and soybean are growing throughout states and years. Qualitatively, we observed that CSDL is less noisy in its classifications than CDL in earlier years (Fig. 1). At the same time, we found that the maps agreed with NASS and ARMS more in some states (Illinois, North Dakota, South Dakota) than others (Kansas, Indiana, Wisconsin), as well as some years than others. CSDL also has a bias toward predicting corn, and tends to under-predict a crop when it is a small fraction of total cropped area in a region. We recommend that users use the metrics provided in our Technical Validation section, which we have released along with the maps, to determine the suitability of CSDL for downstream tasks in a particular region and year.

## Methods

### Pixel-level crop type labels

Creating a crop type map using satellite imagery and supervised machine learning requires field-level ground truth of crop types on which to train a classifier. For CDL, ground truth labels come from the Farm Service Agency’s (FSA) Common Land Unit dataset, which is available to NASS but not to the public due to FSA confidentiality laws^34^. However, the user’s and producer’s accuracy of CDL on FSA labels are displayed in the CDL metadata, and generally exceed 95% for corn and soy from 2008 to 2018. Since we do not have access to FSA data, we used CDL 2008–2018 as our ground truth labels to train our corn and soybeans classifier, and validated our maps using a combination of a CDL 2008–2018 hold-out set, CDL 1999–2007 where it exists, NASS county-level crop acreage estimates, and crop rotation data from ARMS. The year 2008 was chosen to mark the beginning of our training set, because CDL is complete across the conterminous US (and therefore our study region) beginning in that year. The quality of our classifier and validation analyses thus depends on the quality of CDL, NASS, and ARMS data, which we discuss in further detail in the Technical Validation section.

### Cropland mask

Our crop classifier was trained to distinguish between corn, soybeans, and all other crops grouped into a third class; we did not classify cropland from non-cropland. Instead, we used the National Land Cover Database (NLCD) product^35,36^ to mask out non-cropland pixels. NLCD classifies a variety of land cover types — for example, water, developed, and forest — including a single “cultivated crops” class that amalgamates all crop types. The rasters are available at 30 m ground resolution for the years 1992, 2001, 2004, 2006, 2008, 2011, 2013, and 2016. Each year’s CSDL was masked by the last available NLCD product. For example, the 2008–2010 CSDLs are produced with the 2008 NLCD cropland mask. An exception is that the 1999 and 2000 CSDLs use the 2001 NLCD mask, because the previous NLCD product from 1992 was constructed with very different methods.

### Crop acreage statistics

Every year at the end of the harvest season, NASS conducts the County Agricultural Production Survey (CAPS) in cooperation with individual states to estimate acreage and production of selected crops and livestock species at the county level. Each state has its own CAPS sampling strategy, usually involving mail surveys and follow-ups as needed to obtain adequate coverage and response rates^37^. Responses from operators are used to allocate state totals previously obtained from NASS surveys to counties.

We acquired data on the planted area of corn and soybeans at a county level from the NASS Quick Stats database^38^ across the 13 states, and used these county-level acreage estimates as a form of validation on our maps from 1999–2018. Noteworthy for our validation is that, starting in 2009, the CDL program began to be incorporated into the NASS crop acreage estimates through a regression model^4^, so CDL and NASS acreages are not entirely independent datasets. We observed that the number of counties reported in NASS in these 13 states dropped from 1093 in 1999 to 885 in 2018.

### Crop rotation statistics

The Agriculture Resource Management Survey (ARMS), co-sponsored by the USDA’s Economic Research Service and NASS, is a multi-phase series of interviews with farm operators about cropping practices, farm business, and farm households^39^. Within our study period, it was collected in 2001, 2005, and 2010. We used the survey’s crop rotation statistics for the purpose of validating our corn-soybean maps. In each of these three years and for each of the 13 states, we obtained (1) the number of acres that grew corn in the survey year and soybeans in the previous year (a soybean-corn rotation) and (2) the number of acres that underwent a corn-corn rotation. Data were downloaded from the ARMS Tailored Report on Crop Production Practices^40^. We compared the fraction of soybean-corn rotation, defined as $\frac{{\rm{soybean}}\,{\rm{to}}\,{\rm{corn}}\,{\rm{acreage}}}{{\rm{soybean}}\,{\rm{to}}\,{\rm{corn}}\,{\rm{acreage}}+{\rm{corn}}\,{\rm{to}}\,{\rm{corn}}\,{\rm{acreage}}}$, predicted by CSDL against that reported by ARMS.

### Multi-temporal satellite imagery

To perform crop type classification back to 1999, we used annual multi-temporal satellite imagery from the Landsat archive. The Landsat Program is a series of Earth-observing satellites jointly managed by the USGS and NASA, beginning with Landsat-1 in 1972 and continuing with Landsat-7 and -8 in the present day. Its archive was made freely available in 2008^25^, and each satellite offers moderate spatial resolution (30 m) imagery taken every 16 days (every 8 days when two satellites are operating).

**Fig. 2:**
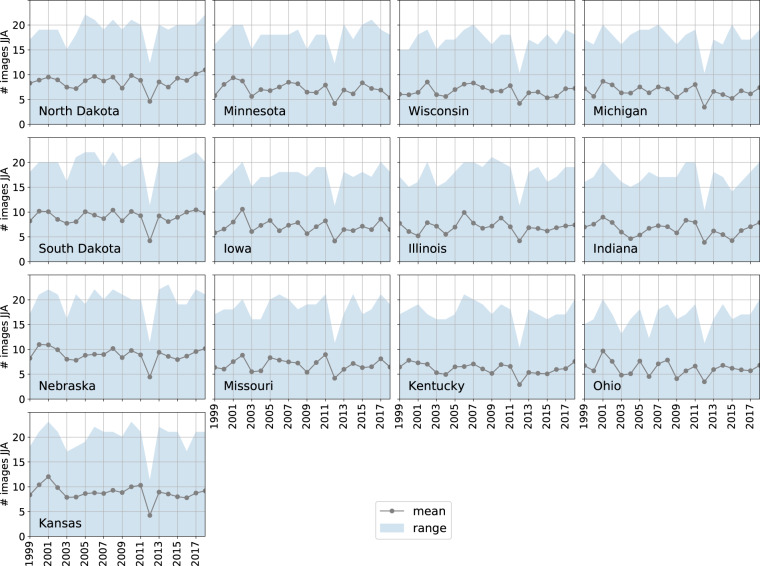
The number of clear Landsat observations available in June, July, and August across states and years. Number of observations were counted for each pixel in our sample after occluded pixels were masked out using the pixel_qa band provided with Landsat Surface Reflectance products. The gray lines show the mean observation availability, while the shading marks the range.

We used Google Earth Engine to obtain imagery over our study region in the period 1999–2018 from Landsat 5, 7, and 8 Surface Reflectance Tier 1 collections^27^. We chose to start in 1999 because Landsat 7 was launched that year. The study region spans 2.2 million km^2^, which corresponds to 2.5 billion Landsat pixels and a total of 87,648 images from January 1, 1999 to December 31, 2018. We used Landsat imagery from January 1 to December 31 to capture crop phenology; in the Midwest, this time window encompasses a single growing season for most crop types. Clouds and other occlusions were masked out at the pixel level using the pixel_qa band that is provided with the Landsat Surface Reflectance products. The median cloud-free image count at a pixel during key crop growing months June–August was 7, with 2012 a notably lower year with a median of 4 (Fig. 2). The dip in 2012 occurred because Landsat 5 Thematic Mapper ended operations in November 2011 and Landsat 8 was not launched until 2013, combined with data gaps from the failed scan-line corrector aboard Landsat 7.

Although the Landsat 5 TM, Landsat 7 ETM+, and Landsat 8 OLI instruments measure slightly different wavelengths due to differing radiometric resolutions, we consider them to share six surface reflectance bands: blue, green, red, near infrared, shortwave infrared 1, and shortwave infrared 2^41^. From these, we derived the green chlorophyll vegetation index (GCVI)^42^,

1 $${\rm{GCVI}}={\rm{NIR}}/{\rm{Green}}-1$$

Unlike NDVI, GCVI does not saturate at high values of leaf area and has previously been shown to aid in distinguishing corn from soybeans^43^. We ultimately included NIR, SWIR1, SWIR2, and GCVI features in our classifier; for an explanation of how these were selected for corn-soybean classification among various bands and vegetation indices, please see ref. ^43^.

### Feature extraction

**Fig. 3:**
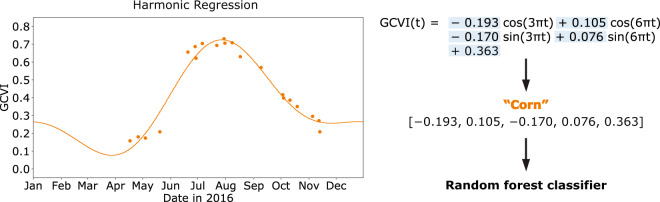
Feature extraction from Landsat time series using a harmonic regression. First, a curve is fit to the Landsat time series (shown here for GCVI) using cosine and sine transformations of the independent variable *t* (time of year). The coefficients and intercept are extracted as features, along with the CDL crop type label, and used to train a random forest classifier.

We summarized the Landsat time series at each pixel using a Fourier transform, or harmonic regression (Fig. 3). Harmonic analysis has previously been shown to successfully capture the different phenology and spectral reflectance across vegetation^44,45^ and crop types^46,47^, allowing corn, soybean, and other crops to be distinguished from each other in the US Midwest^43^. Each spectral band or vegetation index was viewed as a time-dependent function *f*(*t*), so that the harmonic regression takes the form

$$f(t)=c+\mathop{\sum }\limits_{k=1}^{n}\left[{a}_{k}\,\cos (2\pi k\omega t)+{b}_{k}\,\sin (2\pi k\omega t)\right]$$

where *a*_*k*_ are cosine coefficients, *b*_*k*_ are sine coefficients, *c* is the intercept term, *n* is the order of the harmonic series, and *ω* controls the period of the function. The independent variable *t* represents the day of year that an image is taken expressed as a fraction between 0 (January 1) and 1 (next January 1). We used a second order harmonic (*n* = 2) with *ω = *1.5, shown in previous work to result in good features for crop type classification in the study area^43^. This yields a total of 5 features per band or VI (Table 2).

**Table 2:** Features considered in this study.

Source	Name	Details	# of Features	In final map?
Landsat	Blue	Harmonic coefficients	5	N
	Green		5	N
	Red		5	N
	NIR		5	Y
	SWIR1		5	Y
	SWIR2		5	Y
	GCVI		5	Y
gridMET	GDD	Jan 1–Aug 31	1	N
	Mean monthly VPD	Jun, Jul	2	N
	Mean monthly precip	Jun, Jul, Aug	3	N
	Growing season precip	May 1–Sep 15	1	N
	Early season precip	Jan 1–Apr 30	1	N
	Mean monthly max temp	May, Jun, Jul, Aug	4	N
	Mean monthly min temp	May, Jun, Jul, Aug	4	N
	Aridity	Jun 1–Aug 31	1	N
TerraClimate	Climate water deficit	May, Jul	2	N
	Soil moisture	Aug	1	N
Total	—	—	55	20

A total of 55 spectral features and weather covariates were considered during the feature selection stage. The final Corn-Soy Data Layer was created using 20 spectral features.

Our feature extraction procedure therefore requires at least 5 points to fit a second-order harmonic regression and obtain coefficients. The fit will be a better summary of crop phenology if more non-cloudy Landsat images are taken in the growing season.

### Ancillary features

Satellite observations of vegetation are a function of crop type along with plant health and stage of development, which are themselves functions of the amount of sunlight, degree days, water, and nutrients available for growth. We hypothesized that, given measurements of plant phenology via Landsat imagery, observations of these other variables might establish constraints that facilitate the deduction of crop type. To see what we mean, consider a simplified example: suppose we know that a GCVI time series with a max value of 0.7 could be either corn grown under a large number of growing degree days (GDD) or soybean grown under a moderate number of GDD. Then knowing the value of GDD would help us deduce the crop type. We added features that capture some of these weather and climate variables to our classifier and tested to see whether they aid in crop type classification.

We used the University of Idaho’s Gridded Surface Meteorological Dataset (gridMET)^48^ and TerraClimate^49^ to find the growing degree days (GDD), vapor pressure deficit (VPD), mean precipitation, temperature extrema, aridity, soil moisture, and climate water deficit at each pixel in the study region (Table 2). We found that these features did not significantly improve corn and soybean classification in the study region, so our final map is created using only the Landsat-derived harmonic coefficients. However, we note that these ancillary features may still help in a different setting, such as one where weather varies more dramatically across space and time.

### Training set sampling

To sample a set of points that is geographically representative of our study area, we created a 50km-by-50km grid over the 13 states and sampled 250 points uniformly at random from each grid cell. The study area was covered by 839 grid cells; after filtering for points falling inside the boundaries of the 13 states, we were left with a set of 205,821 points. For every year between 1999 and 2018, we used Google Earth Engine to extract (1) the coefficients from harmonic regression fit to the annual Landsat time series at each point and (2) the CDL label at each point if it existed for that year. We removed points from this dataset whose CDL label was nonexistent or a non-crop class, leaving us with 841,028 samples.

A summary of the number of samples for each crop type can be found in Table 3. Across the 13 states, corn is the most numerous class with 329,375 samples, or 39.2% of the total. Soybeans and other crops have 281,411 (33.5%) and 230,215 (27.4%) samples, respectively. The distribution of the three classes varies by state; Iowa, Illinois, Indiana, and Nebraska grew mostly corn and soybean and very little other crop, while North Dakota and Kansas grew mostly other crops.

**Table 3:** Number of samples by state and crop.

Time Period	State	Crop Type	# of Samples	% of Samples
2008–2018	Illinois	Corn	51,336	54.3%
		Soybean	39,458	41.7%
		Other	3,719	3.9%
	Indiana	Corn	25,162	49.0%
		Soybean	23,650	46.0%
		Other	2,549	5.0%
	Iowa	Corn	58,069	55.9%
		Soybean	41,164	39.6%
		Other	4,612	4.4%
	Kansas	Corn	18,522	21.2%
		Soybean	14,349	16.5%
		Other	54,347	62.3%
	Kentucky	Corn	5,358	31.0%
		Soybean	4,861	28.1%
		Other	7,053	40.8%
	Michigan	Corn	11,136	35.0%
		Soybean	9,280	29.2%
		Other	11,404	35.8%
	Minnesota	Corn	34,982	40.4%
		Soybean	33,386	38.5%
		Other	18,261	21.2%
	Missouri	Corn	13,222	30.5%
		Soybean	20,404	47.1%
		Other	9,725	22.4%
	Nebraska	Corn	42,231	55.0%
		Soybean	22,167	28.8%
		Other	12,454	16.2%
	North Dakota	Corn	11,963	13.0%
		Soybean	22,408	24.4%
		Other	57,348	62.5%
	Ohio	Corn	15,207	37.3%
		Soybean	20,623	50.6%
		Other	4,904	12.0%
	South Dakota	Corn	24,504	31.6%
		Soybean	21,707	28.0%
		Other	31,389	40.4%
	Wisconsin	Corn	17,710	46.5%
		Soybean	7,954	20.9%
		Other	12,450	32.7%
	Total	Corn	329,375	39.2%
		Soybean	281,411	33.5%
		Other	230,215	27.4%

For each state, the samples were used to train and validate a classifier to distinguish between three classes: corn, soybean, and all other crops. The crop type labels were derived from CDL in the period 2008–2018. Samples labeled as not cropland were excluded.

Eighty percent of the samples from 2008–2018 were placed in the training set, while the remaining 20% of 2008–2018 and the labeled samples from 1999–2007 were placed in the test set.

### Classification algorithm

**Fig. 4:**
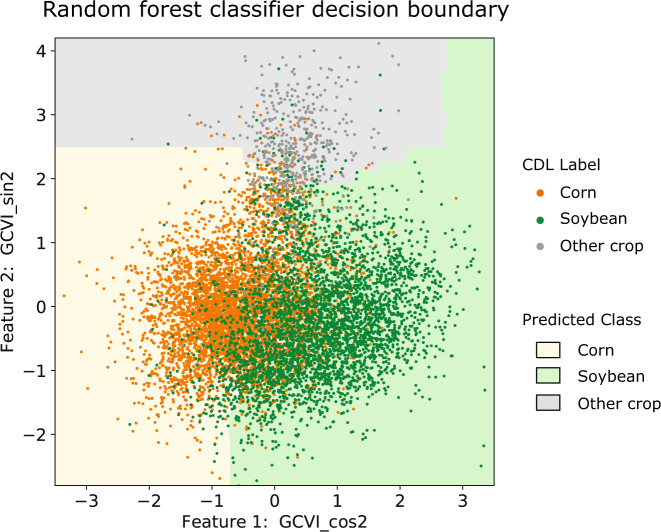
An illustration of how a random forest classifier learns to distinguish among corn, soybean, and other crops in a two-dimensional feature space. Here the features used for classification and visualization are the second order cosine and sine terms of GCVI, and the data points were the ones we exported from Iowa in 2018. Note that the decision boundary learned for our final set of features (20 harmonic coefficients total) would be more complex.

We used random forests for classification, as they are well-documented in the field of remote sensing to perform well on land cover and crop type tasks^50,51^, and usually better than maximum likelihood classifiers, support vector machines, and other methods for crop type mapping^52–55^. Random forests are an ensemble machine learning method comprised of many decision trees in aggregate^56^, and offer ease of use, high performance, and interpretability at the same time. Throughout method development, we used Python’s scikit-learn implementation of a random forest classifier with 500 trees and otherwise default parameters. To create the Corn Soy Data Layer product, we used Google Earth Engine’s ee.Classifier.randomForest, similarly with 500 trees and otherwise default parameters. Figure 4 shows an example decision boundary learned by a random forest to classify corn, soybean, and other crops in Iowa, illustrating the possibility of distinguishing among the three classes.

### Final map creation

To create the Corn-Soy Data Layer (CSDL)^57^, we first used Google Earth Engine to compute the harmonic features for Landsat NIR, SWIR1, SWIR2, and GCVI across the entire study region for each year in 1999–2018. We then used our training samples from 2008–2018 to train a random forest classifier (ee.Classifier.randomForest) on harmonic features to predict crop type labels derived from CDL (corn, soybean, or other). A separate model was trained for each state. Lastly, we applied each classifier to predict crop type on all pixels in the relevant state from 1999–2018.

### Evaluation metrics

In evaluating our CSDL product against CDL, NASS, and ARMS data, we used the following metrics.

First, CSDL and CDL were compared in each state and year using the overall accuracy metric. Accuracy is a commonly used metric for classification and is defined as the fraction of correct predictions. While CDL itself is not a perfectly accurate ground truth of crop type, the accuracy metric computed between CSDL and CDL tells us how closely the two datasets agree. Note that an interpretation of accuracy must take into account the distribution of crop types in an area, as a more skewed distribution biases accuracy upward. To aid interpretation, crop type distributions by state are provided in Table 3.

Second, we computed the user’s accuracy and producer’s accuracy for each crop type in each state and year, using CDL again as “ground truth” for CSDL. If we let TP_*c*_ stand for the number of true positives for crop type *c*, FP_*c*_ the number of false positives, and FN_*c*_ the number of false negatives, then the user’s and producer’s accuracies for crop type *c* are defined as

$$\begin{array}{ccc}{\rm{user\mbox{'}s}}\,{{\rm{accuracy}}}_{c} & = & \frac{{{\rm{TP}}}_{c}}{{{\rm{TP}}}_{c}+{{\rm{FP}}}_{c}}\\ {\rm{producer\mbox{'}s}}\,{{\rm{accuracy}}}_{c} & = & \frac{{{\rm{TP}}}_{c}}{{{\rm{TP}}}_{c}+{{\rm{FN}}}_{c}}\end{array}$$

User’s accuracy is also known as precision, and producer’s accuracy is also known as recall. We have included these metrics with the CSDL map product to aid users in their assessment of how to use CSDL for their applications, and for congruency with the CDL product, which reports user’s and producer’s accuracy in each state’s metadata.

Lastly, to compare aggregated CSDL or CDL to county-level NASS data or state-level ARMS data, we used the coefficient of determination (*R*^2^) metric. The coefficient of determination is defined as

$${R}^{2}=1-\frac{{\sum }_{i}{({y}_{i}-{\widehat{y}}_{i})}^{2}}{{\sum }_{i}{({y}_{i}-\bar{y})}^{2}}$$

where *y*_*i*_ are “ground truth” areas (NASS or ARMS), ${\widehat{y}}_{i}$ are the area predictions under the classifier (CSDL or CDL), and $\bar{y}$ is the mean of ground truth areas. In words, *R*^2^ measures the proportion of the variance in captured by the predictions.

Because we care about absolute error in CSDL predictions, the *R*^2^ between CSDL and NASS is computed *not* for a simple linear regression between *y* and $\widehat{y}$, but on *y* and $\widehat{y}$ values themselves. In this case, *R*^2^ is bounded in the interval (−∞, 1]. It is possible for an *R*^2^ to be negative if the $\widehat{y}$ are worse predictions for *y* than the sample mean $\bar{y}$. In order for *R*^2^ to be close to 1, the predictions must be both positively correlated with ground truth *and* unbiased. *R*^2^ equals 1 only if ${\sum }_{i}{({y}_{i}-{\widehat{y}}_{i})}^{2}=0$, which requires ${y}_{i}={\widehat{y}}_{i}$ for all *i*.

## Data Records

The Corn-Soy Data Layer is available on Google Earth Engine at https://code.earthengine.google.com/?asset=projects/lobell-lab/us_croptype_hindcast/CSDL and for download from Zenodo at 10.5281/zenodo.3742743^57^.

Classes in the CSDL map product are {0: unclassified, 1: corn, 5: soybean, 9: other crop, 255: non-crop}. Note that, for ease of use, these are the same numeric values assigned by CDL to each land cover category. Unclassified pixels may be due to not being in the study region or not having enough Landsat images to fit a harmonic regression. The CSDL product shares the same projection as CDL to facilitate user transition between the two products.

## Technical Validation

### Pixel-level comparison with CDL

**Fig. 5:**
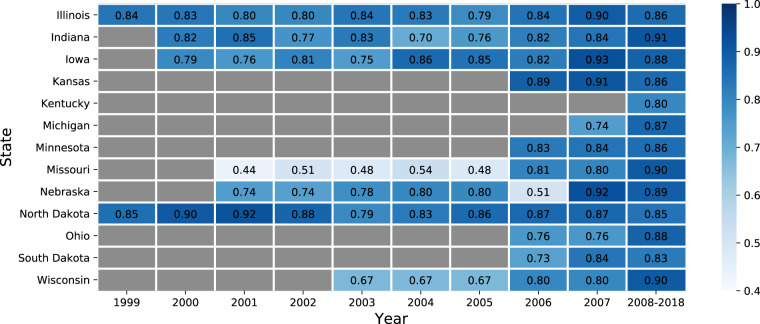
Pixel-level agreement between crop type classified by Corn-Soy Data Layer (CSDL) and crop type classified by CDL. Agreement is measured using accuracy, defined as the fraction of pixels with the same label under both classifications. Dark gray state-years indicate when no CDL classification was available.

First, we compared our maps at the pixel level for sampled points against CDL where it exists for years 1999–2007 and for a hold-out set of sampled points in 2008–2018 (Fig. 5). The percent of pixels classified with the same crop type (corn, soybean, or other) under both maps generally exceeded 85% across states in 2008–2018. This agreement is lower in the test years 1999–2007, with a range from 44% to 93% depending on the state and year. Of the states with long histories of CDL, Illinois, Indiana, Iowa, and North Dakota show high CSDL and CDL agreement, while Missouri 2001–2005 and Nebraska 2006 show low agreement. Though not displayed in this manuscript, we have included a file containing the user’s accuracy (precision) and producer’s accuracy (recall) for each crop type and state along with the CSDL product. These are the same metrics provided in the CDL meta-data.

While some of the mismatch between CSDL and CDL is no doubt due to inaccuracies in the CSDL classifier, some of it can also be attributed to errors in CDL. Qualitatively, our maps appear less noisy in 1999–2007 than CDL where it exists. Figure 1 shows the crop cover classification around Webster City, Hamilton County, Iowa in 2001 for CDL and CSDL. The field-level classifications are the same, despite CDL 2001 being trained on FSA data from 2001 and CSDL being trained on CDL from 2008–2018. However, the fields in CSDL are much more homogeneous, possibly due to the sparing use of Landsat imagery back in 2001 versus our use of all available Landsat 5 and 7.

### County-level comparison with NASS crop acreage statistics

**Fig. 6:**
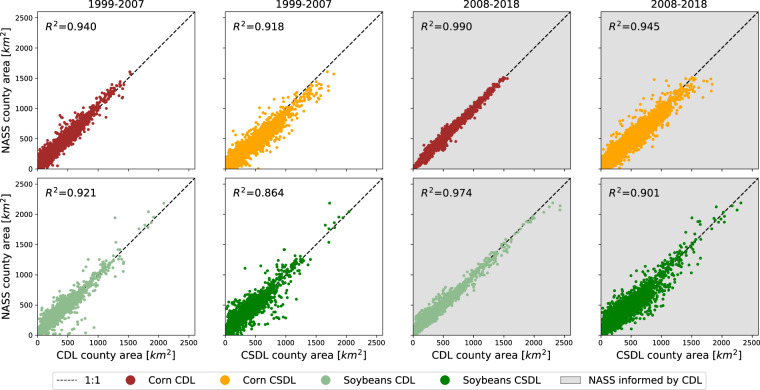
County-level agreement between NASS planted area and CDL or Corn-Soy Data Layer (CSDL) predicted area for corn and soybean. Agreement is measured by the coefficient of determination, *R*^2^, across 1175 counties in the study region. Test years (1999–2007) are shown on the left and training years (2008–2018) are shown with a gray background on the right.

While CDL is only available for all states after 2008, NASS statistics on corn and soybean area planted exist back to the 1970s for counties across the Midwest. Figure 6 shows the coefficient of determination *R*^2^ between annual NASS county area and annual CSDL county area, divided into the test years 1999–2007 and training years 2008–2018. The *R*^2^ between NASS and CDL county areas are also shown for reference. We reiterate that, in order for the coefficient of determination *R*^2^ to be close to 1, the aggregated CSDL or CDL predictions have to be both positively correlated with NASS data *and* unbiased; i.e. the absolute error must be small.

The results in Fig. 6 show that the Corn-Soy Data Layer is able to capture most of the variation in corn and soybean area across counties and years. This is true for the training years 2008–2018 (*R*^2^ = 0.943 for corn, *R*^2^ = 0.901 for soybeans) as well as for the test years 1999–2007, though slightly lower (*R*^2^ = 0.913 for corn, *R*^2^ = 0.865 for soybeans). Agreement between NASS and CDL areas (where available 1999–2007) are overall even higher, and especially so in 2008–2018, when CDL became an input to NASS statistics. Relative to CDL, CSDL predictions show both a slightly larger variance and a bias toward over-predicting corn. Note that, from 1999–2007, the CSDL *R*^2^ is reported across 60 more state-year combinations than the CDL *R*^2^.

**Fig. 7:**
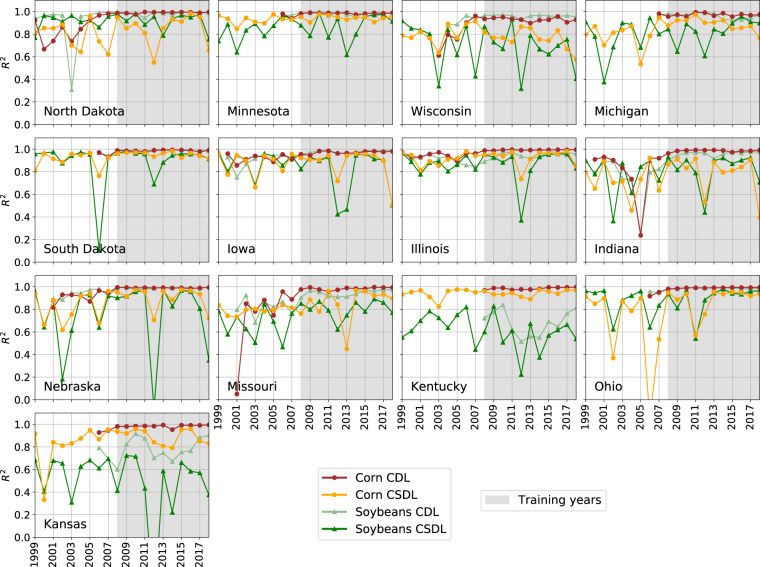
Annual agreement between NASS county-level area and CSDL-predicted county-level area for each state in the study region. Agreement is measured by the coefficient of determination, *R*^2^. Years with a gray background display results in our training years (2008–2018). The order of states corresponds to their relative geographic locations.

While agreement between CSDL and NASS crop area is high across the study area as a whole, a large amount of heterogeneity becomes apparent once the counties are disaggregated to state-years. Figure 7 shows time series of the *R*^2^ between CSDL and NASS county-level planted acreage for each state. Some states have consistently high *R*^2^ across years (Illinois, Iowa, South Dakota, Minnesota), while others have consistently low or variable agreement (Wisconsin, Missouri, Kansas, Ohio). Some years (2013–2017) also have consistently high *R*^2^ across states, while others (2006, 2012) do not. Corn areas usually agreed more with NASS statistics than soybean areas, with North Dakota being an exception with high soybean *R*^2^. In general, the crop types, states, and years with low pixel-level accuracy when compared with CDL corresponded to low *R*^2^ when compared with NASS.

Meanwhile, CDL and NASS areas exhibited high *R*^2^ after 2008 for all states (except soybeans in Kansas and Kentucky). This may be explained by the high quality of FSA data, the use of more than just Landsat satellite images, and CDL informing NASS statistics from 2009 onwards. Prior to 2008, CDL agreement with NASS varies by crop and year for the mapped states, from *R*^2^ s above 0.80 in Iowa and Illinois to below 0.40 in Indiana 2005 and Missouri 2001.

**Fig. 8:**
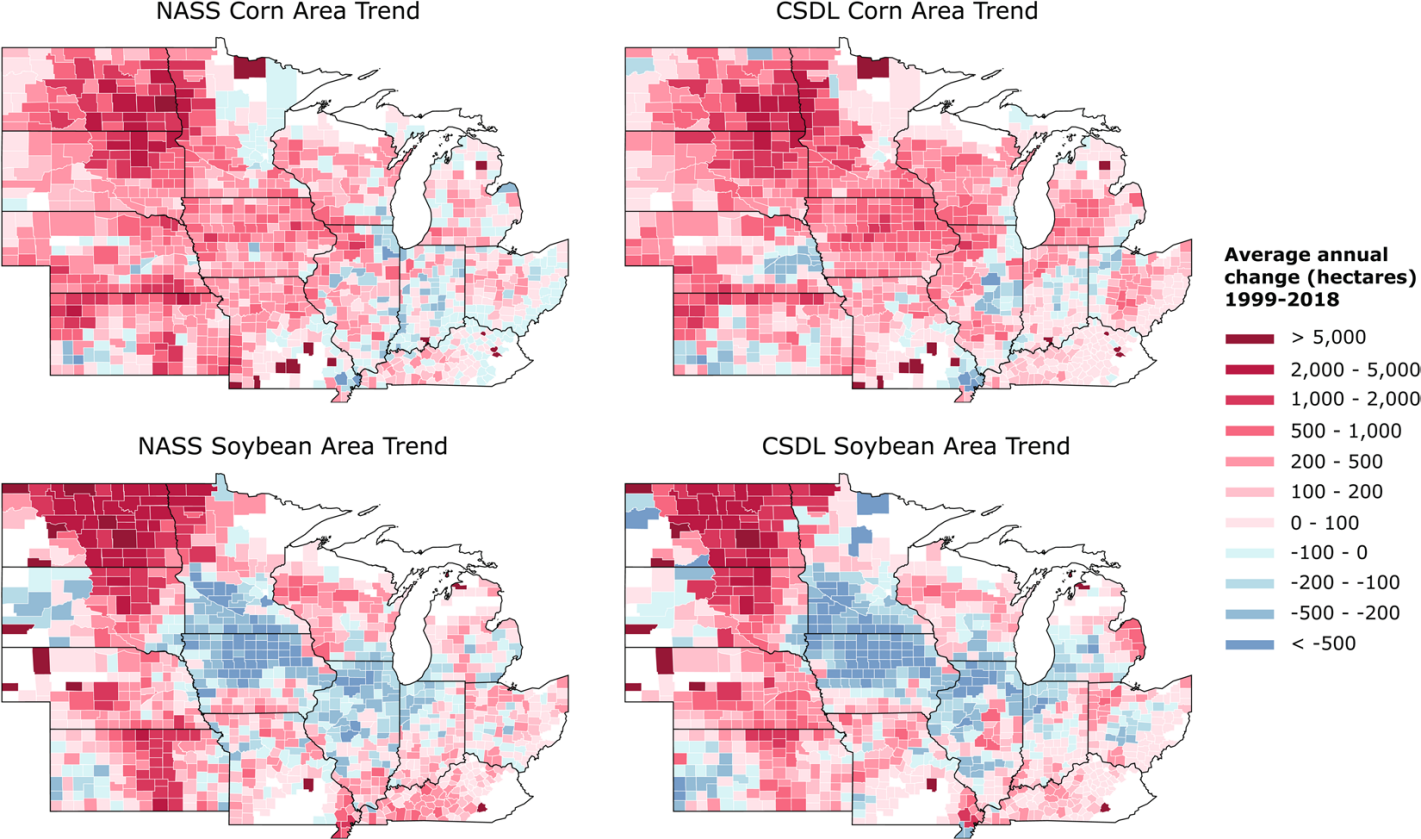
Trends in county-level corn and soybean area from 1999–2018, as measured by NASS or Corn-Soy Data Layer (CSDL). The trend is measured by the slope of the linear regression fit to planted area versus year. Counties in red saw an average annual increase in acreage between 1999 and 2018, while counties in blue saw a decrease.

Looking within each county over time, we found that CSDL planted area can sometimes be biased relative to NASS, but nevertheless shows similar trends from 1999–2018 (Fig. 8). Both NASS and CSDL capture the fact that, in two decades, corn expanded throughout the Midwest, especially in the eastern Dakotas and western Minnesota. Soybean expanded as well, largely in the western states, and contracted in southern Minnesota, northern Iowa and Illinois.

If historical CDL agreement with NASS can be used as a threshold for acceptable quality, then the CSDL fills in many missing years between 1999–2008 in Minnesota, Wisconsin, Michigan, South Dakota, Kentucky, Ohio, and Kansas. However, we did notice that CSDL under-predicted corn or soybean in counties where the crop is a small fraction of total cropped area. We advise users of CSDL to verify the product in a county (for example, against historical NASS) before using it in cross-year analyses.

### State-level comparison with ARMS crop rotation statistics

**Fig. 9:**
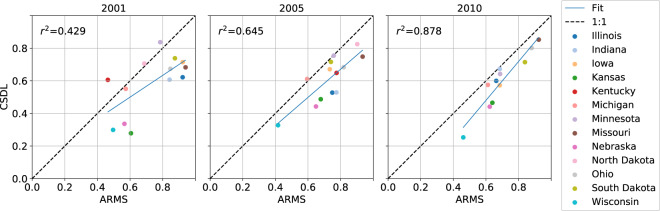
Agreement between ARMS and CSDL-derived rotation statistics for the 13 states in our study region. The statistic being compared is the fraction of corn area in 2001, 2005, or 2010 planted with soybean in the previous year. The squared correlation (*r*^2^) between ARMS and CSDL is shown for each year.

For a verification independent of NASS and CDL, we compared the soybean-to-corn rotation area at the state level derived from CSDL against available ARMS statistics. Figure 9 shows the two datasets’ estimates of the fraction of corn area in 2001, 2005, and 2010 that was previously growing soybean in 2000, 2004, and 2009, respectively. Across the 13 states, the squared correlation (*r*^2^) between CSDL and ARMS estimates improves over time, from 0.421 in 2001, to 0.643 in 2005, to 0.870 in 2010 (though North Dakota and Kentucky are omitted in 2010 because their ARMS data did not report corn-corn rotations). Relative to ARMS, CSDL underestimates the soybean-corn rotation fraction, which is consistent with observations that CSDL overestimates corn and underestimates soybean compared to NASS. Similar to the NASS analyses above, these results suggest that CSDL, like CDL, improves in quality over time, and that CSDL should be assessed for each state and year before used for downstream applications.

### Sources of error in CSDL

Errors in CSDL can be grouped into three main categories of causes: (1) missing or erroneous input data, (2) similarity in corn and soybean spectral reflectance and phenology, and (3) systematic differences between training years (2008–2018) and test years (1999–2007).

Missing or bad input data include limited Landsat imagery (especially during the growing season of May to September), errors in CDL labels, and mislabeling of cropland introduced by our use of NLCD. Landsat image availability may be lowered by clouds or, in the case of 2012, only one Landsat mission being active. There is a noticeable dip in the mean number of clear Landsat images available in 2012 (Fig. 2), and significant areas in each state for which few or no clear images were acquired during key points in the growing season. Crop type classification at these locations became error-prone or impossible, and CSDL classification accuracy compared to CDL and agreement with NASS were correspondingly low in 2012 for many states. Failure of the Landsat 7 scan line corrector in 2003 also caused more gaps in coverage and introduced visible striping artifacts into CSDL 2012. These artifacts are not present in other years when Landsat 5 or 8 are also operational.

**Fig. 10:**
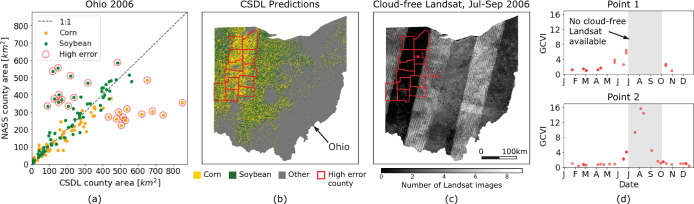
One explanation for CSDL classification failure. Error analysis of CSDL in Ohio 2006 (corn *R*^2^ = −0.17) reveals that (**a**) corn is greatly over-predicted in 12 counties, while soybean is under-predicted. (**b**) These 12 high-error counties are clustered around the center of one Landsat orbit track. (**c**) This Landsat track failed to acquire any clear images between July 1 and September 30, 2006. (**d**) Comparison of a time series inside the track (point 1) and outside it (point 2) shows how peak vegetation was missed and led to the misclassification of much soybean as corn.

Similarly, some individual state-years that have low *R*^2^ in Fig. 7, such as Ohio 2006 and South Dakota 2006, can also be attributed to patches of missing Landsat imagery. For example, Fig. 10 shows that the high errors in Ohio 2006 can be attributed to the absence of any clear Landsat imagery in western Ohio from July 1 to September 30, 2006, which resulted in missing the peak of vegetation. Since soybeans have high GCVI, missing the peak caused soybean pixels to be incorrectly classified as corn or other crop. A potential remedy for the dearth of Landsat imagery would be to include imagery from other satellites, e.g. ResourceSat, the way CDL does.

On the label side, since the CSDL random forest is trained on CDL, the classifier may perpetuate CDL errors, though in an attenuated way if CDL errors from 2008–2018 are not perfectly correlated. During model development, we tried training a classifier on only high confidence CDL labels (>90% confidence band value), but did not observe greater classification accuracy or agreement with NASS. We also do not classify non-cropland ourselves, instead using the most up-to-date NLCD product available in a given year to mask out non-cropland. This results in the years between NLCD updates having out-of-date cropland masks by up to two years. Since cropland expanded from 1999 to 2018, using the last available NLCD should on average result in slight underestimates of cropland area (with the exception of 1999 and 2000, which should be slight overestimates). At the same time, previous studies have shown that cropland expansion in the Midwest has occurred largely on marginal land with severe limits to cultivation^19^, so any error of corn or soybean area should translate to a smaller error in potential downstream production applications.

The second major source of error is that corn and soybean may look similar in spectral reflectance and phenology in some regions and years. To illustrate this, one can imagine that greater similarity translates to the corn points and the soybean points overlapping more in Fig. 4; this makes it difficult for the classifier to distinguish the two. This is true in Nebraska 2002, when both corn and soybeans reached lower-than-average peak GCVI, possibly due to higher-than-average VPD in summer 2002. Since soybean usually has a high GCVI peak in the training set, depressed soybean growth resulted in soybean being misclassified as corn. Previous work also showed that, while soybean is planted and harvested on average later than corn across the Midwest, this difference is more pronounced in the southern states (Illinois, Iowa, Indiana) than the northern ones (Michigan, Wisconsin)^43^. This suggests that classification using Landsat time series would perform better in the southern states, which overall we did observe. CDL also reports lower user’s and producer’s accuracies in the northern states. Overcoming this challenge would require the engineering or acquisition of better, more discerning features.

Lastly, long-term changes or anomalies in climate and crop cultivation could render the statistical associations found between spectral time series and crop type in the period 2008–2018 weaker or inapplicable in 1999–2007. One long-term trend can be found in steadily rising yields; average corn yield between 1999–2007 was 143 bu/ac, while between 2008–2018 it was 161 bu/ac^38^. It is possible that a model trained to differentiate corn and soybean in more recent years would struggle with lower-yielding counterparts in earlier years. Unusual climatic conditions in the test years not observed during training years could also cause errors at test time. For example, unusually heavy precipitation in April and May 2002 delayed planting in Indiana and Ohio; since corn is usually planted earlier than soybean, this shift in planting date caused corn to be misclassified as soybean in these states in 2002. With access to annual FSA data for training, CDL is unaffected by these shifts, but any hindcast model must be wary of them. Improving out-of-domain generalization remains an active area of machine learning research.

## Usage Notes

We recommend that users consider metrics such as *R*^2^ with NASS statistics across space and time to determine in which states/counties and years CSDL is of high quality. This can be done with the annual county-level statistics and example code we have included in our repo at https://github.com/LobellLab/csdl. We have also included, for states and years with CDL, a CSV file summarizing the user’s accuracy (precision) and producer’s accuracy (recall) of CSDL compared against CDL.

## Data Availability

Code used to generate the training samples, compute harmonics, train a random forest classifier, create the final maps, and produce the validation analyses are available publicly as Google Earth Engine scripts, R markdown files, or Jupyter notebooks. Links to scripts and data for analyses can be found in the GitHub repository at https://github.com/LobellLab/csdl. The software used in this work include: • R version 3.5.1, dplyr 0.8.0.1, sf 0.6–3, raster 2.6–7, rgdal 1.3–4, salustools 0.1.0, sp 1.3–1 • Python 3.7.3, numpy 1.16.4, pandas 0.24.2, matplotlib 3.1.0, sklearn 0.21.2, plotly 4.5.0

## References

[1] Food and Agriculture Organization of the United Nations. FAO Statistical Databases, http://faostat.fao.org (2017).

[2] USDA National Agricultural Statistics Service. Cropland Data Layer, https://nassgeodata.gmu.edu/CropScape (2019).

[3] Lark TJ, Mueller RM, Johnson DM, Gibbs HK (2017). Measuring land-use and land-cover change using the u.s. department of agriculture’s cropland data layer: Cautions and recommendations. International Journal of Applied Earth Observation and Geoinformation.

[4] Boryan C, Yang Z, Mueller R, Craig M (2011). Monitoring us agriculture: the us department of agriculture, national agricultural statistics service, cropland data layer program. Geocarto International.

[5] Becker-Reshef I, Vermote E, Lindeman M, Justice C (2010). A generalized regression-based model for forecasting winter wheat yields in kansas and ukraine using modis data. Remote Sensing of Environment.

[6] Bolton DK, Friedl MA (2013). Forecasting crop yield using remotely sensed vegetation indices and crop phenology metrics. Agricultural and Forest Meteorology.

[7] Lobell DB, Thau D, Seifert C, Engle E, Little B (2015). A scalable satellite-based crop yield mapper. Remote Sensing of Environment.

[8] Johnson DM (2014). An assessment of pre- and within-season remotely sensed variables for forecasting corn and soybean yields in the united states. Remote Sensing of Environment.

[9] Franch B (2019). Remote sensing based yield monitoring: Application to winter wheat in united states and ukraine. International Journal of Applied Earth Observation and Geoinformation.

[10] Stern A, Doraiswamy PC, Hunt ER (2012). Changes of crop rotation in Iowa determined from the United States Department of Agriculture, National Agricultural Statistics Service cropland data layer product. Journal of Applied Remote Sensing.

[11] Plourde JD, Pijanowski BC, Pekin BK (2013). Evidence for increased monoculture cropping in the central united states. Agriculture, Ecosystems &. Environment.

[12] Yan L, Roy D (2016). Conterminous united states crop field size quantification from multi-temporal landsat data. Remote Sensing of Environment.

[13] Johnson DM (2013). A 2010 map estimate of annually tilled cropland within the conterminous united states. Agricultural Systems.

[14] Azzari (2019). Satellite mapping of tillage practices in the north central us region from 2005 to 2016. Remote Sensing of Environment.

[15] Hively W, Duiker S, McCarty G, Prabhakara K (2015). Remote sensing to monitor cover crop adoption in southeastern pennsylvania. Journal of Soil and Water Conservation.

[16] Seifert CA, Azzari G, Lobell DB (2018). Satellite detection of cover crops and their effects on crop yield in the midwestern united states. Environmental Research Letters.

[17] Deines JM, Kendall AD, Hyndman DW (2017). Annual irrigation dynamics in the u.s. northern high plains derived from landsat satellite data. Geophysical Research Letters.

[18] Wright CK, Wimberly MC (2013). Recent land use change in the western corn belt threatens grasslands and wetlands. Proceedings of the National Academy of Sciences.

[19] Lark TJ, Salmon JM, Gibbs HK (2015). Cropland expansion outpaces agricultural and biofuel policies in the united states. Environmental Research Letters.

[20] Meehan TD, Werling BP, Landis DA, Gratton C (2011). Agricultural landscape simplification and insecticide use in the midwestern united states. Proceedings of the National Academy of Sciences.

[21] Hendricks NP (2014). The environmental effects of crop price increases: Nitrogen losses in the u.s. corn belt. Journal of Environmental Economics and Management.

[22] Maalim F, Melesse A, Belmont P, Gran K (2013). Modeling the impact of land use changes on runoff and sediment yield in the le sueur watershed, minnesota using geowepp. Catena.

[23] Bennett AB, Isaacs R (2014). Landscape composition influences pollinators and pollination services in perennial biofuel plantings. Agriculture, Ecosystems & Environment.

[24] Johnson DM (2019). Using the landsat archive to map crop cover history across the united states. Remote Sensing of Environment.

[25] Wulder MA, Masek JG, Cohen WB, Loveland TR, Woodcock CE (2012). Opening the archive: How free data has enabled the science and monitoring promise of landsat. Remote Sensing of Environment.

[26] Claverie M, Vermote EF, Franch B, Masek JG (2015). Evaluation of the landsat-5 tm and landsat-7 etm+ surface reflectance products. Remote Sensing of Environment.

[27] Gorelick N (2017). Google earth engine: Planetary-scale geospatial analysis for everyone. Remote Sensing of Environment.

[28] Lark, T. J. *et al*. Impacts of the U.S. Renewable Fuel Standard - Linking Policy, Economics, and Environmental Outcomes. In *AGU Fall Meeting Abstracts*, vol. 2019, GC21C–1268 (2019).

[29] Seifert CA, Roberts MJ, Lobell DB (2017). Continuous corn and soybean yield penalties across hundreds of thousands of fields. Agronomy Journal.

[30] Deines JM, Wang S, Lobell DB (2019). Satellites reveal a small positive yield effect from conservation tillage across the US corn belt. Environmental Research Letters.

[31] Mekonnen MM, Hoekstra AY, Neale CM, Ray C, Yang HS (2020). Water productivity benchmarks: The case of maize and soybean in nebraska. Agricultural Water Management.

[32] Shrestha R (2017). Regression model to estimate flood impact on corn yield using modis ndvi and usda cropland data layer. Journal of Integrative Agriculture.

[33] Bhattarai MD, Secchi S, Schoof J (2017). Projecting corn and soybeans yields under climate change in a corn belt watershed. Agricultural Systems.

[34] Johnson DM, Mueller R (2010). The 2009 cropland data layer. Photogrammetric Engineering and Remote Sensing.

[35] Yang L (2018). A new generation of the united states national land cover database: Requirements, research priorities, design, and implementation strategies. ISPRS Journal of Photogrammetry and Remote Sensing.

[36] Homer C (2020). Conterminous united states land cover change patterns 2001–2016 from the 2016 national land cover database. ISPRS Journal of Photogrammetry and Remote Sensing.

[37] USDA National Agricultural Statistics Service. County Agricultural Production, https://www.nass.usda.gov/Surveys/Guide_to_NASS_Surveys/County_Agricultural_Production/index.php (2019).

[38] USDA National Agricultural Statistics Service. Quick Stats, https://quickstats.nass.usda.gov/ (2019).

[39] USDA Economic Research Service. ARMS Farm Financial and Crop Production Practices, https://www.ers.usda.gov/data-products/arms-farm-financial-and-crop-production-practices/documentation/ (2018).

[40] USDA Economic Research Service. ARMS Farm Financial and Crop Production Practices Tailored Reports: Crop Production Practices, https://data.ers.usda.gov/reports.aspx?ID=17883 (2019).

[41] Roy D (2014). Landsat-8: Science and product vision for terrestrial global change research. Remote Sensing of Environment.

[42] Gitelson, A. A., Vina, A., Ciganda, V., Rundquist, D. C. & Arkebauer, T. J. Remote estimation of canopy chlorophyll content in crops. *Geophysical Research Letters* **32** (2005).

[43] Wang S, Azzari G, Lobell DB (2019). Crop type mapping without field-level labels: Random forest transfer and unsupervised clustering techniques. Remote Sensing of Environment.

[44] Moody A, Johnson DM (2001). Land-surface phenologies from avhrr using the discrete fourier transform. Remote Sensing of Environment.

[45] Scharlemann JPW (2008). Global data for ecology and epidemiology: A novel algorithm for temporal fourier processing modis data. Plos One.

[46] Jakubauskas ME, Legates DR, Kastens JH (2002). Crop identification using harmonic analysis of time-series avhrr ndvi data. Computers and Electronics in Agriculture.

[47] Mingwei Z (2008). Crop discrimination in northern china with double cropping systems using fourier analysis of time-series modis data. International Journal of Applied Earth Observation and Geoinformation.

[48] Abatzoglou JT (2013). Development of gridded surface meteorological data for ecological applications and modelling. International Journal of Climatology.

[49] Abatzoglou JT, Dobrowski SZ, Parks SA, Hegewisch KC (2018). Terraclimate, a high-resolution global dataset of monthly climate and climatic water balance from 1958–2015. Scientific Data.

[50] Gislason, P. O., Benediktsson, J. A. & Sveinsson, J. R. Random forests for land cover classification. *Pattern Recognition Letters* **27**, 294–300 (2006). Pattern Recognition in Remote Sensing (PRRS 2004).

[51] Azzari, G. & Lobell, D. Landsat-based classification in the cloud: An opportunity for a paradigm shift in land cover monitoring. *Remote Sensing of Environment* **202**, 64–74 (2017). Big Remotely Sensed Data: tools, applications and experiences.

[52] Ok AO, Akar O, Gungor O (2012). Evaluation of random forest method for agricultural crop classification. European Journal of Remote Sensing.

[53] Inglada J (2015). Assessment of an operational system for crop type map production using high temporal and spatial resolution satellite optical imagery. Remote Sensing.

[54] Gomez C, White JC, Wulder MA (2016). Optical remotely sensed time series data for land cover classification: A review. ISPRS Journal of Photogrammetry and Remote Sensing.

[55] Ghazaryan G (2018). A rule-based approach for crop identification using multi-temporal and multi-sensor phenological metrics. European Journal of Remote Sensing.

[56] Breiman L (2001). Random forests. Machine Learning.

[57] Wang S, Di Tommaso S, Deines J, Lobell D (2020). Zenodo.

